# Efficacy and Long-Term Safety of *H. pylori* Eradication for Gastric Cancer Prevention

**DOI:** 10.3390/cancers11050593

**Published:** 2019-04-28

**Authors:** Jyh-Ming Liou, Yi-Chia Lee, Emad M. El-Omar, Ming-Shiang Wu

**Affiliations:** 1 Division of Gastroenterology and Hepatology, Department of Internal Medicine, National Taiwan University Hospital, Taipei 10002, Taiwan; jyhmingliou@gmail.com (J.-M.L.); yichialee@ntu.edu.tw (Y.-C.L.); 2 Department of Internal Medicine, National Taiwan University College of Medicine, Taipei 10002, Taiwan; 3 Department of Internal Medicine, National Taiwan University Cancer Center, Taipei 106, Taiwan; 4 Microbiome Research Centre, St George & Sutherland Clinical School, University of New South Wales, Sydney, NSW 2217, Australia

**Keywords:** *H. pylori*, screening, eradication, gastric cancer prevention, efficacy, safety

## Abstract

*Helicobacter pylori (H. pylori)* has been shown to be a causal factor of gastric cancer in cohort studies and animal models. Meta-analysis of case-control studies nested within prospective cohorts showed that *H. pylori* infection was associated with a 5.9-fold increased risk of non-cardia gastric cancer. Prospective cohort studies showed that gastric cancer developed in 1–4% of *H. pylori-*infected subjects. Gastric cancer was successfully induced in Mongolian gerbils and insulin-gastrin (INS-GAS) transgenic mice after inoculation of *H. pylori*. Meta-analysis of randomized control trials also showed that eradication of *H. pylori* may reduce the risk of gastric cancer. However, there are several concerns regarding the widespread use of antibiotics to prevent gastric cancer, including the emergence of antibiotic resistance and the perturbation of gut microbiota after *H. pylori* eradication. Recent studies showed that eradication of *H. pylori* resulted in an increase in the bacterial diversity and restoration of the relative abundance of other bacteria to levels similar to *H. pylori* non-infected subjects in the gastric microbiota. The administration of antibiotics may also alter the composition of intestinal microbiota. The α-diversity and β-diversity of fecal microbiota are significantly altered immediately after *H. pylori* eradication but are gradually restored to levels similar to those before therapy. Yet, the rate of recovery varies with regimens. The diversity was restored at week 8 after triple therapy but was not yet fully recovered at 1 year after concomitant and quadruple therapies. Some studies showed that supplementation of probiotics may reduce the dysbiosis during *H. pylori* eradication therapy. Although some earlier studies showed high levels of macrolide resistance after triple therapy, recent studies showed that the increased antibiotic resistance rate may be restored 2–12 months after eradication therapy. These results collectively provide evidence of the long-term safety of *H. pylori* eradication. Yet, more prospective cohort studies and randomized trials are warranted to assess the efficacy and long-term safety of *H. pylori* eradication for gastric cancer prevention.

## 1. Literature Search

Gastric cancer remains the third most common cause of cancer-related mortality worldwide [1]. It is estimated that about 750,000 people die of gastric cancer each year [1,2]. Early detection of gastric cancer through a nationwide endoscopic/radiographic screening program has been conducted in Japan to reduce the mortality rate of gastric cancer [3,4]. However, the incidence of gastric cancer remains high despite such secondary prevention programs [3,4]. Therefore, primary prevention through the elimination of its etiological factor is a promising strategy to reduce the incidence of gastric cancer. In this article, we will discuss the feasibility and potential concerns of mass screening and eradication of *Helicobacter pylori (H. pylori)* to prevent gastric cancer. We searched PubMed using the terms “gastric cancer”, “risk factors”, “*H. pylori*”, “eradication”, “prevention”, “gut microbiota”, “antibiotic resistance”, “body weight”, “gastroesophageal reflux disease”, “metabolic”, and “insulin resistance”, for papers published up to 31 December 2018, without language restrictions. The references of related articles were also searched. 

## 2. Risk Factors of Gastric Cancer 

**Figure 1:**
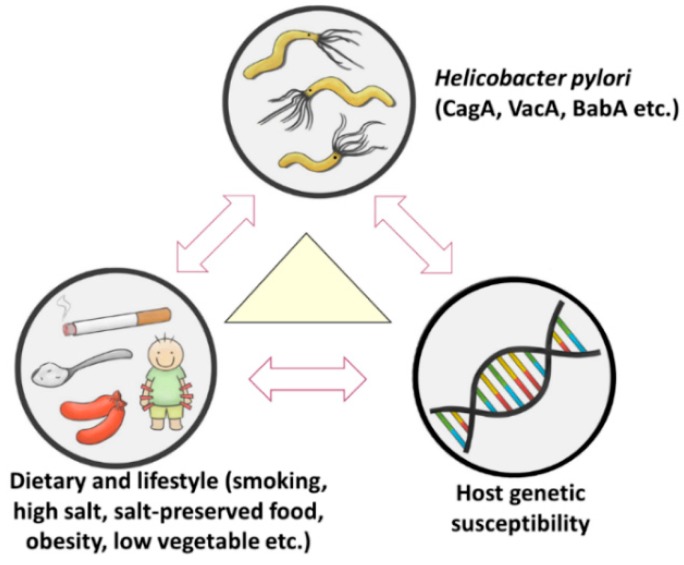
Risk factors of gastric cancer. Interaction of dietary and lifestyle factors, host genetic factors, and *H. pylori* infection contribute to gastric carcinogenesis.

Gastric cancer is a heterogeneous and multi-factorial disease [4,5]. Interaction of dietary and lifestyle factors, host genetic factors, and *H. pylori* infection contribute to gastric carcinogenesis (Figure 1) [4,5].

### 2.1. Lifestyle and Dietary Factors

Excessive dietary salt intake, preserved or fried foods are associated with and increased risk of gastric cancer [6,7]. A prospective cohort study including 2500 subjects in Japan showed that a high salt diet was an independent risk factor of gastric cancer after adjustment of *H. pylori* and atrophic gastritis. Intake of fresh fruit and vegetable is associated with reduced risk of gastric cancer [6]. Systematic review and meta-analysis of cohort studies showed that current cigarette smoking was associated with increased risk of gastric cancer, compared with never smoking for both cardia (relative risk (RR) 1.87, 95% confidence interval (CI) 1.31–2.67) and non-cardia (RR 1.60, 95% CI 1.41–1.80) cancers [8]. A systematic review and meta-analysis of 24 studies showed that being overweight (body mass index (BMI), 25–30 kg/m^2^) and obesity (BMI, ≥30 kg/m^2^) are associated with increased risk of gastric cardia cancer (summary RR 1.21 for overweight and 1.82 for obesity), but not non-cardia gastric cancer [9]. The association between alcohol consumption and gastric cancer remains controversial, although some studies reported increased risk of distal cancer for those who drank more than 15 g of alcohol per day, compared with non-drinkers [10,11].

### 2.2. Host Genetic Factors

There is a trend of familial aggregation of gastric cancer, indicating the importance of host genetic factors in the carcinogenesis of this tumor [12]. Host genetic factors can be divided into high, intermediate, and low penetrance genes [12,13,14]. The penetrance rate of germline mutations E-cadherin ranges between 50% to 70% for hereditary diffuse gastric cancer, which accounts for 1–3% of gastric cancers [12,15,16]. The penetrance rates of germline mutations at *hMLH1* or *hMSH2* for gastric cancer are intermediate (2–30%), accounting for less than 10% of gastric cancers [12]. The penetrance rates of genetic polymorphisms in pro-inflammatory genes (*IL1B, IL8, TNFA*, etc.) and *PSCA* (Prostate Stem Cell Antigen) are low (probably less than 5%) for sporadic gastric cancer, which accounts for more than 90% of gastric cancer [12,13,14].

### 2.3. Helicobacter Pylori Infection

*H. pylori*, a gram-negative bacillus, was identified from patients with peptic ulcer disease by Barry Marshall and Robin Warren in 1982 [17]. Subsequent human studies and animal models showed that *H. pylori* is a causal risk factor for gastric cancer [18,19,20,21,22,23,24].

#### 2.3.1. Observational Human Studies

Ecological association studies have shown a positive correlation of seroprevalence rate of *H. pylori* with local gastric cancer incidence and mortality rates in 13 European countries including 3194 subjects (Table 1) [18]. Meta-analysis of 12 nested case-control studies including 1228 gastric cancer cases and 3406 controls showed that *H. pylori-*infected subjects have a 3-fold increased risk of non-cardia gastric cancer [19]. Subgroup analysis of studies that used blood samples collected more than 10 years before the diagnosis of gastric cancer further showed that *H. pylori*-infected subjects had a 5.9-fold increased risk of non-cardia gastric cancer [19]. In a prospective cohort study including 1246 *H. pylori* infected and 280 non-infected Japanese subjects followed for 7.8 years, gastric cancer developed in 2.9% (36/1246) vs. 0% (0/280) among *H. pylori-*infected and non-infected subjects, respectively [20]. Another prospective cohort study in Taiwan showed that gastric cancer developed in 1.1% (7/618) vs. 0% (0/607) among *H. pylor*i-infected and non-infected subjects, respectively, after a median follow-up for 6.3 years [21]. The results provide an important message that gastric cancer rarely develops in patients without the presence of *H. pylori* at baseline.

#### 2.3.2. Animal Models

Animal studies showed that gastric cancer developed in 37% (10/27) of Mongolian gerbils 62 weeks after inoculation of *H. pylori* and in 75% (6/8) of insulin-gastrin (INS-GAS) transgenic mice after inoculation of *H. felis* (Table 2) [22,23]. Lee et al. and Romero-Gallo et al. showed that early eradication of *H. pylori* may reduce the risk of severe dysplasia or gastric cancer in mice and Mongolian gerbils, respectively (Table 2) [24,25].

## 3. Interaction of Lifestyle, Dietary, Host Genetic Factors and *H. pylori* in Gastric Carcinogenesis 

**Figure 2:**
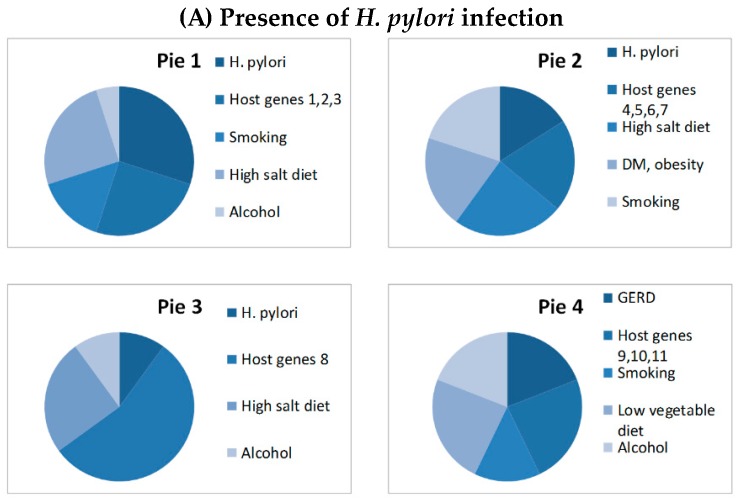

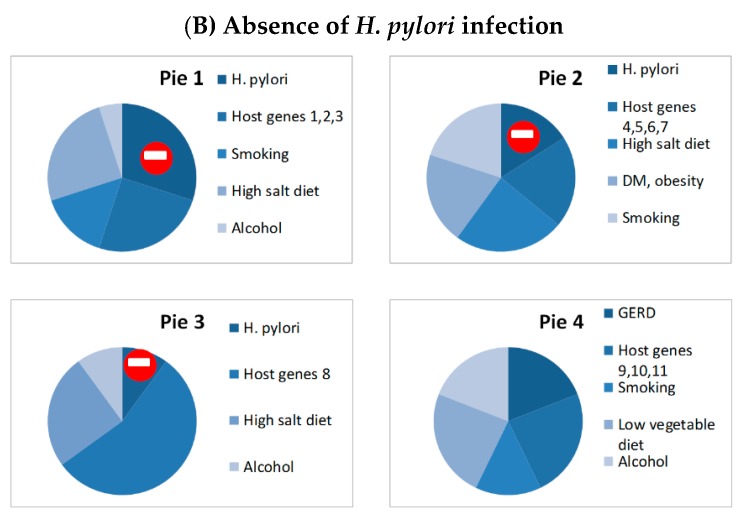
The causal pie model—role of *H. pylori* in gastric carcinogenesis. *H. pylori*: *Helicobacter pylori*; DM: diabetes mellitus; GERD: gastroesophageal reflux disease.

Gastric cancer develops in only 1–4% of *H. pylori* infected subjects, indicating that *H. pylori* alone is not sufficient to cause gastric cancer [20,21]. Therefore, we used the causal pie model to explain the interactions of *H. pylori* with lifestyle/dietary factors and host genetic factors in gastric carcinogenesis (Figure 2). Causal pie model has been proposed to explain the sufficient causes and component causes of multifactorial diseases [26]. A disease may have several sufficient causes that are composed of by several component causes [27]. The disease will develop when all necessary components of the sufficient cause (termed as a causal pie) are present [27]. The etiologies of gastric cancer can also be explained by the causal pie model (Figure 2). There are several sufficient causes of gastric cancer. If a *H. pylori*-infected subject with host genetic factors 1, 2, 3 has the lifestyle of smoking, alcohol drinking and taking high salt diet, gastric cancer will develop eventually, assuming he/she has not died of other competing causes (pie 1). However, gastric cancer will not develop if any one of the necessary components is not present in pie 1. Although *H. pylori* is a necessary component of several sufficient causes of gastric cancer (pie 1, 2, and 3), some subtypes of gastric cancer are not attributed to *H. pylori* (pie 4). Gastric cancer may develop eventually if all of the necessary components in pie 4 are present. Epidemiological studies suggested that the attributable fraction of *H. pylori* for gastric cancer is about 74.7–89%, indicating that 70% of gastric cancer can be prevented if *H. pylori* is removed [28]. Eradication of *H. pylori* may reduce the risk of gastric cancer, but the magnitude of risk reduction depends on the timing of eradication. Certain genetic alterations may occur with aging and chronic inflammation caused by *H. pylori* infection, leading to atrophic gastritis and intestinal metaplasia. At this stage, gastric cancer may develop even if *H. pylori* is removed [29].

## 4. Can *H. pylori* Eradication Reduce the Risk of Gastric Cancer?

Several cohort studies and randomized trials have assessed the effect of *H. pylori* eradication on the risk of gastric cancer or the risk of progression of gastric precancerous lesions [26,29,30]. In a recent systematic review and meta-analysis of cohort studies and randomized trials, Lee et al. identified 6 randomized trials and 8 cohort studies recruiting asymptomatic infected individuals for primary prevention of gastric cancer [26]. They also identified two randomized trials and 8 cohort studies recruiting patients with early gastric cancer that assessed the risk of synchronous gastric cancer after curative endoscopic resection [30]. Meta-analysis of all studies showed that eradication of *H. pylori* reduced the incidence of gastric cancer, compared with those not receiving eradication therapies (pooled incidence rate ratio 0.53, 95% CI 0.44–0.64) [26]. In the subgroup analysis, the pooled incidence rate ratios were 0.60 (95% CI: 0.44–0.81) and 0.52 (95% CI: 0.41–0.64) for randomized trials and cohort studies, respectively [26]. Recently, another randomized trial from Korea also showed that eradication of *H. pylori* in patients with early gastric cancer may reduce the risk of synchronous gastric cancer after curative endoscopic resection [31]. These findings collectively support that eradication of *H. pylori* may reduce the risk of gastric cancer. However, there were some limitations of the above studies, including the relative small sample size, the use of progression of precancerous lesions as the primary outcome in many studies, and lack of data from non-East Asian populations. A search of clinical trial registration databases showed that there are several ongoing large randomized trials comparing the efficacy of eradication therapy versus no eradication therapy for gastric cancer prevention in *H. pylori* infected subjects from China, Korea, and Latvia (Table 3). Another large placebo-controlled randomized trial was conducted in the United Kingdom to compare the efficacy of eradication therapy vs. no eradication in the primary prevention of peptic ulcer bleeding in chronic aspirin users (Table 4). It is expected that the results from these large randomized trials will provide more robust evidence on this issue. 

## 5. Can Screening and Eradication of *H. pylori* Reduce the Risk of Gastric Cancer?

**Figure 3:**
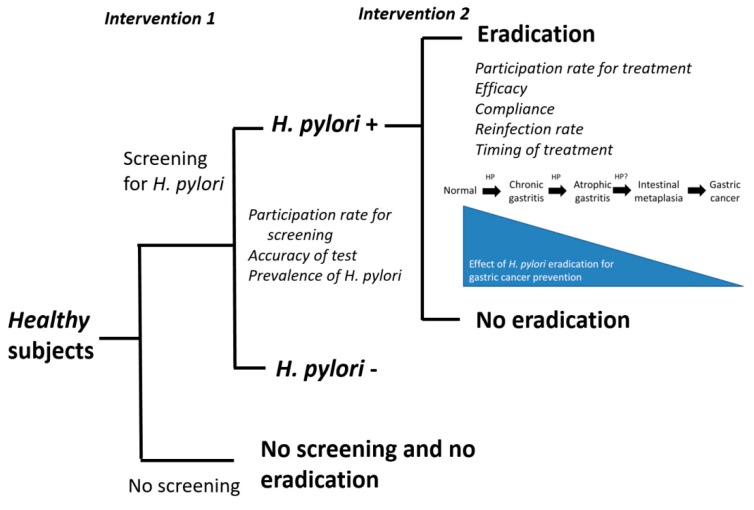
Factors affecting the efficacy of the strategy of screening and eradication of *H. pylori* for gastric cancer prevention. *H. pylori*: *Helicobacter pylori*; HP: *Helicobacter pylori*.

Another important issue is whether mass screening and eradication of *H. pylori* is effective for primary prevention of gastric cancer (Figure 3) [38]. The optimal study design to address this question is to randomize healthy subjects into one group with screening and eradication of *H. pylori* and the other one without screening and eradication therapy (randomization on intervention 1, Figure 3). However, all of the previous trials and cohort studies addressed the efficacy of eradication therapy (intervention 2) for gastric cancer prevention in *H. pylori* subjects. Many factors should be considered to assess whether screening and eradication of *H. pylori* is effective for gastric cancer prevention in the general population (Figure 3). This strategy is expected to be more effective for gastric cancer prevention if the participation rate for screening and the accuracy of diagnostic tests for *H. pylori* are both high in countries with high prevalence of *H. pylori* infection and high incidence of gastric cancer. Eradication of *H. pylori* is expected to be more effective for gastric cancer prevention if the participation rate for eradication therapy is high, the efficacy and compliance to the therapy are high, the reinfection rate is low, and the therapy is given early before the development of precancerous lesions. A large trial randomizing healthy subjects into one group with screening for *H. pylori* and the other without screening using incidence of gastric cancer as primary outcome is ongoing in Taiwan. The much-awaited results of this trial will provide important evidence on this issue. 

## 6. Other Beneficial Effects of *H. pylori* Eradication

**Figure 4:**
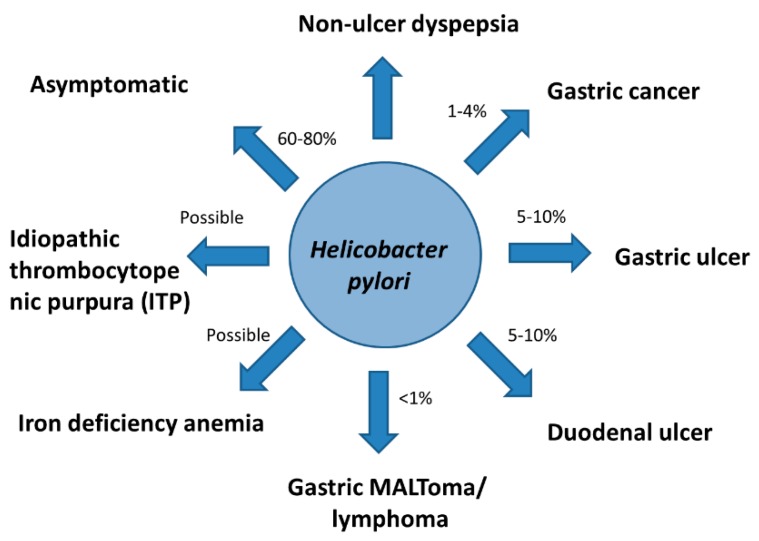
*H. pylori* and human diseases. *H. pylori*: *Helicobacter pylori*; MALToma: mucosa-associated lymphoid tissue lymphoma.

Epidemiological studies showed that gastric ulcer, duodenal ulcer, gastric cancer, and gastric mucosal-associated lymphoid tissue lymphoma (MALToma) develop in 5–10%, 5–10%, 1–4%, and <1% among *H. pylori* infected subjects, respectively (Figure 4) [39,40]. Non-ulcer dyspepsia, and some extra-gastric disorders, such as iron deficiency anemia and idiopathic thrombocytopenic purpura (ITP) are also associated with *H. pylori* infection (Figure 4) [39,40,41,42]. Eradication of *H. pylori* may reduce the risk of gastric cancer, reduce the risk of synchronous gastric cancer after endoscopic resection of early gastric cancer, reduce the risk of recurrent peptic ulcer disease, lead to complete remission of MALToma, and relieve dyspeptic symptoms [39,40,41,42,43]. Meta-analysis of randomized trials showed that eradication of *H. pylori* may reduce the risk of recurrent peptic ulcer and may improve the symptoms of dyspepsia [44,45]. Eradication of *H. pylori* may also lead to complete remission of gastric MALToma in about two-thirds of patients [46]. The complete response rate of ITP defined as platelet count of 100 × 10^9^/L or greater was observed in about 42% of patients [47]. Therefore, international consensus reports recommended that eradication therapy is strongly indicated for *H. pylori* infected patients who have these diseases [40,41,42,43,48].

## 7. Potential Harms or Concerns of Eradication Therapy

**Figure 5:**
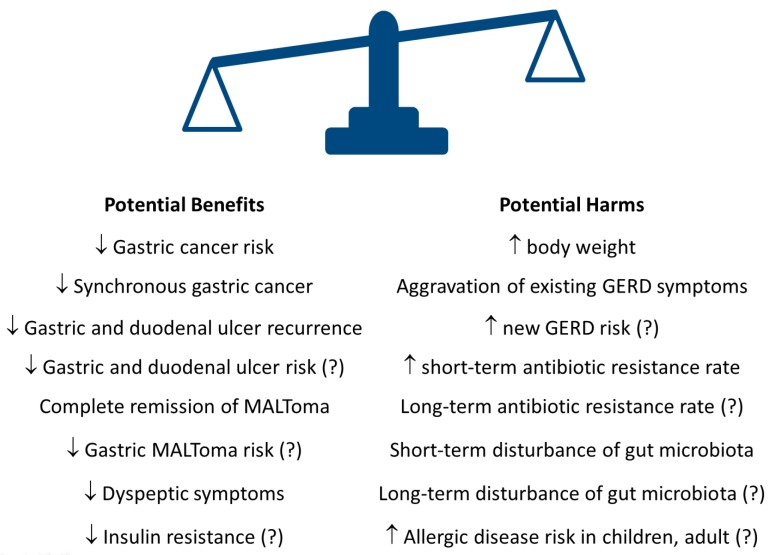
Potential benefits and harms after *H. pylori* eradication therapy. *H. pylori*: *Helicobacter pylori*; MALToma: mucosa-associated lymphoid tissue lymphoma; GERD: gastroesophageal reflux disease.

Although eradication of *H. pylori* may have the potential benefits mentioned above, there are some potential concerns including an increase in antibiotic resistance rates, disturbance of gut microbiota, increase in body weight and aggravation of existing GERD symptoms etc. (Figure 5) [49,50,51,52]. Few studies have addressed the long-term impact of eradication therapy on antibiotic resistance rate and the gut microbiota.

### 7.1. Impact on Antibiotic Resistance

**Figure 6:**
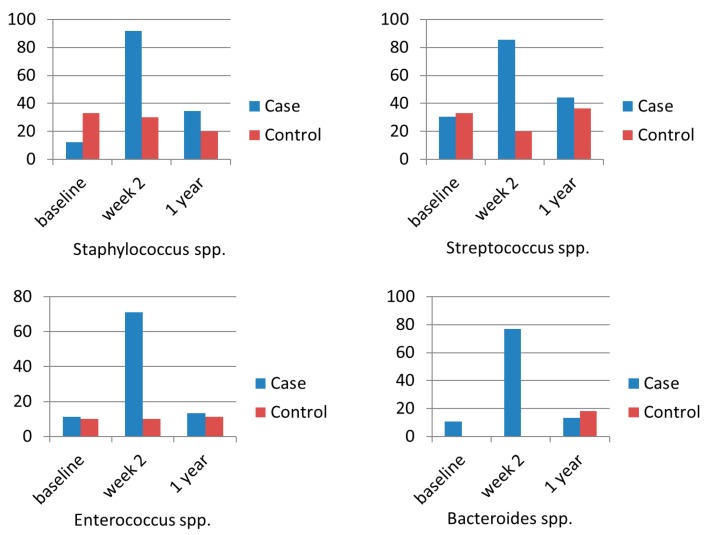
The impact of triple therapy on the antibiotic resistance of clarithromycin [6].

Emergence of antibiotic resistance after widespread use of antibiotics is one of the most important concerns of mass screening and eradication of *H. pylori* for asymptomatic subjects in the community [49]. A case-control study showed that the clarithromycin resistance rate of *Enterococcus* increased from 0% (0/5) to 100% (5/5), compared to 0% (0/5) to 20% (1/5) in the control group immediately after triple therapy [50]. The resistance rates were 50% (2/4) and 0% (0/5) in the *H. pylori* eradication group and control group 1 year after eradication therapy, respectively [50]. In another case control study, Jakobson et al. showed that the clarithromycin resistance rates of *Staphylococcus* spp., *Streptococcus* spp., *Enterococcus* spp., and *Bacteroides* spp. were significantly increased immediately after triple therapy (Figure 6) [51]. The clarithromycin resistance rates were still numerically higher 1 year after therapy than that of baseline, but the differences were not statistically significant (Figure 6) [51]. Our recent randomized trial showed that the antibiotic resistance rates to several antibiotics (penicillin derivatives, cephalosporin, and fluoroquinolones etc.) of *Escherichia coli* were significantly increased immediately after triple therapy and concomitant therapy, but were not significantly changed after bismuth quadruple therapy [52]. However, the transient increases of antibiotic resistant rates were restored to baseline levels one year after eradication therapy [52]. More studies from other ethnic populations and countries are warranted on this issue. Development of novel anti-microbial agents specific for *H. pylori* is another approach to avoid the emergence of antibiotic resistance of other bacterium. Some antimicrobial peptides, such as β-defensins, pexiganan, tilapia piscidins, epinecidin-1, cathelicidins, bicarinalin, and bacteriocins may inhibit the growth of *H. pylori* and have the potential to replace traditional antibiotics for *H. pylori* eradication and gastric cancer prevention [53,54].

### 7.2. Impact on Gut Microbiota

Antibiotics are important modulators of gastric and gut microbiota [55]. In the stomach, there was an inverse association between *H. pylori* and the diversity of gastric microbiota [56]. The use of eradication therapies containing two or more antibiotics may lead to short-term and long-term changes of gastric and gut microbiota [32,33,34,35,36,37,57]. Eradication of *H. pylori* may result in an increase in the diversity of gastric microbiota [57]. The relative abundance of other bacteria in the stomach may be restored to levels similar to individuals without *H. pylori* infection [57]. Eradication therapy for *H. pylori* may also exert short-term alteration of gut microbiota [32,34,35,36,37]. The short-term changes of gut microbiota after triple therapy or bismuth quadruple therapy have been reported in 5 studies [32,34,35,36,37]. All of these studies showed that bacterial diversity was significantly altered immediately after eradication therapy [32,34,35,36,37]. Oh et al. showed that the relative abundance of Firmicutes was reduced whereas that of Proteobacteria was increased immediately after triple therapy [34]. Yanagi et al. showed that the Bacteroidetes/Firmicutes ratio was significantly greater than baseline at week 8 after triple therapy [35]. However, Chen et al. showed that the Bacteroides/Firmicutes ratio was significantly reduced immediately after bismuth quadruple therapy [37]. Hsu et al. showed that the relative abundances of Bacteroidetes and Actinobacteria reduced significantly, whereas that of Proteobacteria increased 8 weeks after bismuth quadruple therapy [36]. However, relatively few studies reported the long-term changes (≥6 months) of gut microbiota after *H. pylori* eradication [35,36,37]. Jakobsson et al. and Hsu et al. showed that the diversity of the microbiota recovered to the pre-treatment status 1 year later, but there were some notable changes at the genus level after triple therapy and bismuth quadruple therapy [32,36]. Yap et al. also showed the α-diversity was restored to baseline levels, there were some notable changes at the phylum and genus levels 18-months after triple therapy [33]. In a multicenter randomized trial, Liou et al. showed that α-diversity and β-diversity were restored to baseline level 12 months after triple therapy, but were not yet fully recovered to baseline 12 months after bismuth quadruple and concomitant therapy [52]. More studies with longer follow-up periods are warranted on this issue.

### 7.3. Impact on Gastroesophageal Reflux Disease (GERD)

Some studies suggested that the risk of GERD might be increased in some patients after *H. pylori* eradication, probably attributed to restoration of gastric acid secretion after elimination of *H. pylori* [58,59,60]. Body weight gain after *H. pylori* eradication may also lead to aggravation of GERD symptoms. However, a systematic review and meta-analysis of 7 randomized trials showed that the risk of erosive esophagitis was not significantly increased in *H. pylori* eradicated group, compared with the persistent *H. pylori* group (odds ratio (OR) 1.11, 95% CI 0.81–1.53) [59]. Meta-analysis of 5 cohort studies also showed that the risk of GERD in *H. pylori* eradicated group was not significantly increased (OR 1.22, 95% CI 0.89–1.69), compared with the persistent *H. pylori* group [59]. There are some explanations for the contradictory results. Earlier studies suggested that gastric acid secretion is increased in *H. pylori* patients with antrum predominant gastritis, whereas it is reduced in *H. pylori* patients with corpus predominant gastritis [61]. The gastric acid secretion might be normalized after improvement of gastritis after *H. pylori* eradication [61]. Thus the gastric secretion would be reduced in those with antrum predominant gastritis and would be increased in those with corpus predominant gastritis. Therefore, the GERD symptoms in *H. pylori-*infected patients might be relieved in patients with antrum predominant gastritis after *H. pylori* eradication but might be aggravated in patient with corpus predominant gastritis. However, this confounding factor was not included in the analysis in most of the previous randomized trials and cohort studies [58,59,60]. These factors should be considered in future trials addressing this issue.

### 7.4. Impact on Body Weight and Metabolic Parameters 

Case-control studies showed an inverse association of *H. pylori* and body weight [62]. Some cohort studies showed an increase of body weight after *H. pylori* eradication [63]. A recent systematic review and meta-analysis showed significant increase of body mass index (BMI) (mean difference 0.36, 95% 0.11–0.60) and body weight (mean difference 1.1kg, 95% CI 0.8–1.5) after eradication of *H. pylori* [63]. In a randomized control trial, 1558 *H. pylori* infected subjects were randomized into eradication therapy and placebo groups [64]. The mean BMI increased from 27.5 to 27.8 kg/m^2^ and 27.0 to 27.2 kg/m^2^ in the eradication group and placebo groups, respectively [64]. The adjusted difference of BMI between the two groups was 0.2 kg/m^2^ (95% CI: 0.11, 0.31) [64]. The increase in body weight is probably attributed to the restoration of ghrelin secretion or the relief dyspeptic symptoms [65]. Yet, the clinical significance of this trivial increase in body weight remains questionable. Some studies showed that insulin resistance, fasting glucose, total cholesterol, and triglyceride levels were reduced after *H. pylori* eradication [52,66]. The changes in these metabolic parameters might be attributed to the alterations in gut microbiota [54]. However, the findings remain controversial and further well-designed randomized trials are warranted to clarify the impact of *H. pylori* eradication on metabolic parameters.

## 8. Endoscopic Surveillance for Gastric Cancer after *H. pylori* Eradication

Another important question is that gastric cancer might still develop in some patients, especially in those with pre-existing intestinal metaplasia and atrophic gastritis [28]. A cohort study showed that the risk of gastric cancer was 0.25% per year among 61,707 patients with intestinal metaplasia in the Dutch nationwide histology register [67]. Although the risk might be even lower after *H. pylori* eradication, it is important to identify subjects at higher risk of gastric cancer and provide surveillance endoscopy for them. Demographic characteristics (age, gender, ethnicity etc.) and the measurement of serum pepsinogen might help us to identify those who need screening endoscopy at the time of *H. pylori* eradication. A multicenter prospective cohort study from The Netherlands and Norway showed that none of the patients with limited atrophic gastritis or intestinal metaplasia (defined as pepsinogen I/II ratio >3 and operative link on gastric intestinal metaplasia (OLGIM) stage 0–II) develop high-grade adenoma/dysplasia or invasive neoplasia during a mean follow-up of 57 months [68]. In contrast, gastric cancer developed in 4 patients with extended atrophic gastritis/intestinal metaplasia (pepsinogen I/II ratio ≤3 or OLGIM stage III–IV) [68].

Little is known about whether the genetic and epigenetic changes at the time of *H. pylori* eradication may be helpful for the risk stratification for surveillance endoscopy. Although the gastric cancer risk can be effectively reduced by *H. pylori* eradication, certain irreversible damages may have occurred after decades of chronic *H. pylori* infection [69]. Methods to quantify these intracellular damages and correlate the levels with the severity of premalignant gastric lesions and the risk of gastric cancer are under enthusiastic evaluation; limited endoscopic resources can, therefore, be allocated efficiently [70]. The success of epigenetic drugs, such as de-methylation agents in the cancer treatment, has indicated that the measurement of *H. pylori*-induced DNA methylation is one of the possible solutions [71]. *H. pylori* infection may lead to an increase of DNA methylation levels of some functional genes while the high methylation levels may persist even after *H. pylori* eradication. In this regard, researchers in Japan have found that the higher methylation levels of *miR-124a-3*, *EMX-1*, and *NKX6-1* in the normally appearing gastric mucosae were associated with the higher risk of metachronous of gastric cancer after patients have received endoscopic resection of early gastric cancer [72,73]. For application in the general population, two prospective studies are ongoing [74,75]; the former was a multicenter study in Japan while the latter was a community-based study in Matsu Islands (Taiwan), where the policy of screening and treating *H. pylori* infection has been implemented for more than one decade [26].

Recently, a comprehensive molecular investigation of the genetic and epigenetic alterations using whole-exome sequencing of gastric biopsy specimens from 148 individuals in the Gastric Cancer Epidemiology Program (GCEP) has been reported [76]. Huang et al. showed the mutations (e.g., *TP53, ARID1A*, and *FBXW7*) commonly detected in gastric cancer were infrequent (<5%) in the biopsy specimens of gastric intestinal metaplasia [76]. Somatic copy number aberrations were 12.5% and 0% in incomplete and complete intestinal metaplasia, respectively. Interestingly, they found that shortened telomeres and chromosomal alterations were associated with subsequent dysplasia or gastric cancer, whereas normal-like epigenomic patterns were associated with regression of intestinal metaplasia [76]. Yet, further validation studies are warranted to assess whether the genetic and epigenetic alterations may be implicated in the risk stratification of surveillance endoscopy after *H. pylori* eradication. 

## 9. Conclusions

More than 70% of gastric cancer can be attributable to *H. pylori*, although only 1–4% of *H. pylori* infected subjects will develop gastric cancer eventually. Eradication of *H. pylori* can reduce the risk of gastric cancer. Yet, more data from ongoing trials are needed to assess whether the incidence of gastric cancer could be reduced by mass screening and eradication of *H. pylori*. There are other potential benefits, such as reduction in the risk of peptic ulcer disease. However, more data are needed to provide evidence for mass screening and eradication of *H. pylori* in the community. There is a short-term increase in the antibiotic resistance rate and alteration of gut microbiota after *H. pylori* eradication. However, the antibiotic resistance rate appears to have been restored 1 year later, especially in those treated with bismuth quadruple therapy. The diversity of gut microbiota can be restored to baseline status 1 year later, although some changes at genus level are not fully recovered. Eradication of *H. pylori* may also impact positively on the metabolic parameters, probably through the alteration of gut microbiota. These results collectively provide evidence of the long-term safety of *H. pylori* eradication. Yet, more prospective cohort studies and randomized trials are warranted to assess the long-term efficacy and safety of *H. pylori* eradication for gastric cancer prevention. 

## Figures and Tables

**Table 1:** *H. pylori* and gastric cancer risk in human.

Study Design	Case Number	Findings
Ecological study [18]	3194 subjects from 13 European countries	Prevalence rates of *H. pylori* correlated with local gastric cancer incidence and mortality rates
Meta-analysis nested case-control studies [19]	12 nested studies including 1228 GC cases and 3406 controls	Non-cardia gastric cancer: OR 3.0; 95% CI: 2.3–3.8 Blood samples collected ≥ 10 years before GC diagnosis: OR 5.9, 95% CI: 3.4–10.3
Prospective cohort study [20,21]	1246 *H. pylori* infected and 280 non-infected Japanese, followed for 7.8 years [20]	GC developed in 2.9% (36/1246) vs. 0% (0/280) among *H. pylori* infected and non-infected subjects, respectively
	618 *H. pylori* infected and 607 non-infected Taiwanese, followed for 6.3 years [21]	GC developed in1.1% (7/618) vs. 0% (0/607) among *H. pylor*i infected and non-infected subjects, respectively
Meta-analysis of intervention trials [26]	24 studies including 715 incident gastric cancers among a total of 48,064 individuals/340,255 person-years	*H. pylori* eradication reduced the risk of GC, with pooled incidence rate ratio of 0.53; 95% CI: 0.44–0.64.

*H. pylori*: *Helicobacter pylori*; GC: gastric cancer; OR: odds ratio; CI: confidence interval.

**Table 2:** *H. pylori* and gastric cancer risk in animal models.

Study	Animal	Findings
Observational		
Watanabe et al. [22]	Mongolian gerbils	GC developed in 37% (10/27) of gerbils 62 weeks after inoculation of *H. pylori.*
Wang et al. [23]	INS-GAS transgenic mice	GC developed in 75% (6/8) of the mice after inoculation of *H. felis*.
Intervention trial		
Lee et al. [24]	INS-GAS transgenic mice	Severe dysplasia and GC developed 28 weeks after inoculation of *H. pylori*. Early eradication of *H. pylori* at 8 weeks after inoculation may prevent the development of dysplasia.
Romero-Gallo [25]	Mongolian gerbils	GC or dysplasia developed in 60% of gerbils with persistent *H. pylori* infection, compared to none in the eradicated group.

*H. pylori*: *Helicobacter pylori*; INS-GAS: insulin-gastrin; GC: gastric cancer.

**Table 3:** Ongoing trials comparing the efficacy of *H. pylori* eradication on the risk of gastric cancer.

Clinical Trial Registration Number	Subjects	Design	Experiment Group	Control Group	Primary Outcome	Estimated Sample Size	Country	Age (Years)
NCT02047994	Healthy *H. pylori* infected subjects	Open label	triple therapy	No treatment	Gastric cancer mortality	30,000	Latvia	40–64
NCT02112214	Healthy *H. pylori* infected subjects	Double blind	bismuth quadruple therapy	Placebo	Gastric cancer incidence	11,000	Korea	40–60
NCT01678027	Sibling or offspring of patients with gastric adenocarcinoma	Double blind	triple therapy	Placebo	Gastric cancer incidence	1,810	Korea	40–65
ChiCTR-TRC-10000979	Healthy residents	Double blind	bismuth quadruple therapy	bismuth + omeprazole + placebo	Gastric cancer incidence	184,786	China	25–54
NCT01506986	*H. pylori-*infected aspirin user	Double blind	triple therapy	Placebo	Peptic ulcer bleeding	33,000	UK	≥60
NCT01741363	Healthy subjects	Open label	*H. pylori* screening and FIT	FIT alone	Gastric cancer incidence	60,000	Taiwan	50–75

FIT: fecal immunochemical test; UK: United Kingdom.

**Table 4:** The impact of *H. pylori* eradication therapy on the gut microbiota (sequencing of 16S rRNA).

Authors, Year	Case Number	Regimen Used for HP Eradication	Immediately After Therapy	Short-Term Changes (2–3 Months)	Long-Term Changes (≥6 months)
Jakobsson et al., 2010 [32]	6	PCA for 7 days	bacterial diversity ↓	N/A	Diversity of the microbiota recovered to resemble the pre-treatment states, but some notable changes at genus levels
Yap et al., 2015 [33]	17	PCA for 7 days			No significant differences in α-diversity and β-diversity; some notable changes at the phylum and genus levels
Oh et al., 2016 [34]	23	PCA +/− probiotics for 7 days	relative abundances of Firmicutes↓, Proteobacteria ↑ in both groups		N/A
Yanagi et al., 2017 [35]	20	PCA for 7 days		Bacteroidetes:Firmicutes (B:F) ratio was significantly greater than baseline	N/A
Hsu et al., 2018 [36]	11	Bismuth quadruple therapy for 10 days		relative abundance of Proteobacteria↑; Bacteroidetes ↓; Actinobacteria↓ at week 8	No significant differences in α-diversity and β-diversity; some notable changes at the phylum and genus levels
Chen et al., 2018 [37]	70	Bismuth quadruple +/− probiotics for 14 days	α-diversity decreased and the B:F ratio decreased from 0.98 to 0.34 on day 14	α-diversity not completely recovered on day 56; B:F ratio increased to 0.83 on day 56.	N/A

PCA: proton pump inhibitor plus clarithromycin and amoxicillin; HP: *Helicobacter pylori;* N/A: not available.
